# Implementation of the Framework Convention on Tobacco Control in Africa: Current Status of Legislation

**DOI:** 10.3390/ijerph8114312

**Published:** 2011-11-17

**Authors:** Jacqueline Tumwine

**Affiliations:** Health and Environmental Rights Organisation (HERO-Uganda), PO BOX 16649, Kampala, Uganda; E-Mail: jt327@law.georgetown.edu

**Keywords:** Framework Convention on Tobacco Control, FCTC, tobacco control policies/interventions, bans on cigarette advertising and promotions, clean-indoor air laws, public smoking bans, smoke-free environments, tobacco industry interference, legislation and jurisprudence, cigarette labeling, Africa

## Abstract

**Objective:**

To describe, as of July 2011, the status of tobacco control legislation in Africa in three key areas of the Framework Convention on Tobacco Control (FCTC)—(1) Protection from exposure to tobacco smoke, (2) Packaging and labelling of tobacco products, and (3) Tobacco advertising, promotion and sponsorship.

**Methods:**

Review and analysis of tobacco control legislation in Africa, media reports, journal articles, tobacco industry documents and data published in the 2011 WHO Report on the Global Tobacco Epidemic.

**Results:**

Modest progress in FCTC implementation in Africa with many countries having legislation or policies on the protection from exposure to tobacco smoke, however, only a handful of countries meet the standards of the FCTC Article 8 and its Guidelines particularly with regards to designated smoking areas. Little progress on packaging and labelling of tobacco products, with few countries having legislation meeting the minimum standards of the FCTC Article 11 and its Guidelines. Mauritius is the only African country with graphic or pictorial health warnings in place and has the largest warning labels in Africa. Slightly better progress in banning tobacco advertising, promotion and sponsorship has been shown by African countries, although the majority of legislation falls short of the standards of the FCTC Article 13 and its Guidelines. Despite their efforts, African countries’ FCTC implementation at national level has not matched the strong regional commitment demonstrated during the FCTC treaty negotiations.

**Conclusion:**

This study highlights the need for Africa to step up efforts to adopt and implement effective tobacco control legislation that is fully compliant with the FCTC. In order to achieve this, countries should prioritise resources for capacity building for drafting strong FCTC compliant legislation, research to inform policy and boost political will, and countering the tobacco industry which is a major obstacle to FCTC implementation in Africa.

## 1. Background

Tobacco use kills 5.4 million people worldwide each year and if current trends continue, this figure is set to reach 8.3 million by the year 2030, with 80% of these tobacco related deaths occurring in developing countries [1]. Sub-Saharan Africa is in the early stages of the tobacco epidemic, characterised by low rates of smoking for women and men, but increasing popularity of cigarettes among men [2]. Tobacco use in Africa is on the increase as the tobacco industry shifts its marketing focus from the West to “areas of strong growth” of Africa and Asia [3]. This is worrying as the fragile health systems in many African countries are unable to adequately cope with the double burden of infectious diseases and chronic diseases including those that are caused by tobacco use.

The WHO Framework Convention on Tobacco Control (WHO FCTC) was a global response to the globalisation of the tobacco epidemic [4]. The treaty was adopted in May 2003 by the World Health Assembly and entered into force in February 2005. The FCTC requires state Parties to adopt and implement tobacco control measures including but not limited to the following: packaging and health warning labels on tobacco products; bans on tobacco advertising, promotion and sponsorship; measures to protect people from tobacco smoke; tobacco tax and price increases; regulation of the contents of tobacco products; regulation of tobacco product disclosure; support for economically viable alternatives; measures to curb illicit trade in tobacco products; liability provisions and others. As of July 2011, the FCTC has 168 signatories and 174 Parties. Out of the 46 countries in the WHO African region, 41 are Parties to the FCTC [5]. Africa’s commitment to the FCTC was evident during the FCTC negotiations process as countries in the region rallied with a unified voice in support of strong treaty provisions [6]. It is important to assess whether Africa’s FCTC commitment has translated into effective national implementation of the treaty.

This paper aims to describe, as of July 2011, the status of tobacco control legislation in Africa in three key areas of the Framework Convention on Tobacco Control: (1) protection from exposure to tobacco smoke (Article 8); (2) packaging and labelling of tobacco products (Article 11); and (3) tobacco advertising, promotion and sponsorship (Article 13). These three areas were chosen as a focus for the paper because of their centrality in the tobacco control campaigns in Africa and also because they were the first three articles of the FCTC to have guidelines adopted for their implementation thus facilitating analysis of legislation across the continent to measure compliance with the FCTC.

The paper will analyse the extent to which various legislation in Africa complies with the FCTC and the Guidelines for the implementation of the FCTC which are based on scientific evidence and best practice [7]. The paper will also identify obstacles to FCTC implementation in Africa as well as opportunities to improve the status quo.

## 2. Methods

Data published in the 2011 WHO Report on the Global Tobacco Epidemic (which is based on country-level reporting using a standardised questionnaire) was analysed to identify the African countries’ general progress in the areas of protection from exposure to tobacco smoke, packaging and labelling of tobacco products, and tobacco advertising, promotion and sponsorship. In addition, texts of tobacco control legislation of African countries were collected, reviewed and analysed in conjunction with the texts of the Framework Convention on Tobacco Control and the three sets of Guidelines for the implementation of FCTC Articles 8, 11, and 13. The legislative analysis and review was necessary to determine whether the legislation was fully compliant with the standards of the FCTC and its Guidelines. Collection of legislation was done by carrying out online searches using Google search engine. Key words used in the searches included a combination of a country’s name plus either one of the following phrases: “tobacco control act”; “smoking act”; “tobacco control regulations”; “smoking regulations”; “loi antitabac”; and “arrêté antitabac”. Legislation was also obtained from tobacco control advocates in Africa upon request.

Supplementary information was collected from the following data sources: journal articles (using PubMed search, keywords: Africa, FCTC, tobacco); tobacco industry internal documents (using Legacy Tobacco Documents Library search, keywords: advertising, marketing, health warnings, smoking, ban, bill, legislation, Cora, Amesca AND Africa); and media reports (using Google search, keywords were same as the aforementioned ones used to search country legislation). The supplementary information was reviewed and analysed to identify the obstacles to FCTC implementation in Africa and the opportunities to advance FCTC implementation.

## 3. Results and Discussion

### 3.1. Protection from Exposure to Tobacco Smoke (FCTC Article 8)

#### 3.1.1. Current Status of Legislation and Compliance with the FCTC

The 2011 WHO Report on the Global Tobacco Epidemic published data on, among other things, the global status of smoke-free legislation covering eight types of public places: health care facilities; educational facilities; universities; public transport; government facilities; indoor offices; restaurants; and pubs and bars [8]. According to the report, only four countries in the African region have all eight public places completely smoke-free (or at least 90% of the population covered by complete sub-national smoke-free legislation) [9]. Three countries have a ban on smoking in six to seven types of public places [10]. Eight countries have three to five types of public places completely smoke-free [11], while a total of thirty countries had between zero and two types of public places that are completely smoke-free [12].

Upon analysis of smoke-free legislation in Africa by the authour of this paper, an overwhelming majority of countries (91.3%) was found to have legislative provisions that permit designated smoking rooms. Designated smoking rooms are in violation of the FCTC Article 8 and the FCTC Article 8 Guidelines which require “effective” measures of protection from exposure to tobacco smoke. As envisioned by Article 8 of the FCTC, “effective” measures require the total elimination of smoking and tobacco smoke in a particular space or environment in order to create a 100% smoke free environment [13]. Moreover, the Article 8 Guidelines provide that Article 8 of the FCTC creates an *obligation* to provide *universal protection* by ensuring that all indoor public places, all indoor workplaces, all public transport, and appropriate outdoor or quasi-outdoor public places are free from exposure to tobacco smoke [14]. Furthermore, scientific evidence shows that there is no safe level of exposure to tobacco smoke and that approaches other than 100% smoke free environments, including ventilation, air filtration and the use of designated smoking areas (whether with separate ventilation systems or not), have repeatedly been shown to be ineffective and there is conclusive evidence, scientific and otherwise, that engineering approaches do not protect against exposure to tobacco smoke [15].

Namibia bans smoking in public places and makes no exception for smoking in designated smoking areas thereby creating 100% smoke-free environments and fully meeting the standards of the FCTC and its Guidelines [16]. Chad also bans smoking in public places without exception for designated smoking areas [17]. Chad further prohibits smoking in vehicles that have a minor or pregnant person on board [18]. Seychelles bans smoking in public places, workplaces and public transport without designated smoking rooms, however, the law does not apply to hotel rooms although the owner may prohibit or restrict smoking in such a bedroom [19].

Mauritius almost meets the FCTC Article 8 Guidelines with regards to 100% smoke-free environments. The law in Mauritius bans smoking in public places (cafés, bars, nightclubs and restaurants) without provisions of designated smoking areas. However, the law’s shortcoming is that designated smoking areas are permitted in workplaces, which contravenes the FCTC Article 8 and Article 8 Guidelines [20]. With regards to banning smoking in outdoor or quasi-outdoor places, the legislation in Mauritius conforms to the FCTC as smoking is banned in outdoor areas of the following: health institutions, educational institutions, sports and recreational places other than beaches to which the public has access, bus stations and bus stands [20].

Although South Africa’s smoke-free legislation allows for designated smoking areas [21], the country has made headway with regards to banning smoking in outdoor and quasi outdoor areas. Recent amendments to South Africa’s smoke-free law have extended the areas where smoking is prohibited to include: partially enclosed areas; areas within a prescribed distance from windows, doorways or entrances of public places; and any prescribed outdoor public place where persons are likely to congregate within close proximity of one another or where smoking may pose a fire or other hazard. The amendments made South Africa the first country in the world to have a nationwide ban prohibiting smoking in cars where persons below 12 are present [22]. South Africa also took steps to ensure a smoke-free FIFA World Cup in 2010 [23]. This is an example of how opportunities to host international or regional events can be used by African countries to boost national smoke-free efforts.

While not perfect, South Africa’s success in tobacco control can be attributed to the political system. South Africa, in the post-apartheid era, saw a shift from a pro-tobacco government to one that had the political will to advance tobacco control. Under the new political leadership of the Africa National Congress (ANC) South Africa made important tobacco control advances including legislation banning public smoking and banning tobacco advertising, with the help of Health Ministers supportive to the tobacco control cause [24].

In the absence of comprehensive national tobacco control legislation passed by parliament, some African countries have smoke-free ministerial directives. These are administrative rules issued by a specific Ministry to regulate smoking in specified locations under that Ministry’s authority. Ghana has ministerial directives that regulate smoking in government buildings, health and education facilities, and public transport [25]. However, enforcement of these directives is difficult as they lack legal backing [26]. Senegal has ministerial directives prohibiting smoking in all premises of the Ministry of Health and in education institutions. In addition, there is a smoke-free municipal by-law in Touba [27]. Enacting sub-national legislation like in Senegal’s case has helped fill the gap in several African countries that do not have national tobacco control legislation but whose systems of government permit legislating at sub-national level. Such countries besides Senegal include Central African Republic (Bangui), the Islamic Federal Republic of the Comoros (Automous Island of Ngazidja) and the Federal Republic of Nigeria (Osun State and the Federal Capital Authority-Abuja).

While sub-national smokefree legislation may not offer protection to the whole population, legislating at local, state, provincial or municipal levels can be more effective especially in countries where the tobacco industry wields more power at national levels than at sub-national levels [28]. Furthermore it may be more feasible to pursue sub-national smokefree legislation where there happens to be less political will and financial and human resources at national level than at sub-national level to pass and enforce smoke-free legislation.

Although enacting sub-national smoke-free legislation in Africa is encouraged, countries must ensure that such legislation complies with the FCTC and the FCTC guidelines in order to offer effective protection against tobacco smoke. In addition, where national legislation is enacted, pre-emption clauses should be avoided, that is, clauses that prohibit sub-national or local legislation that is stronger or stricter than the national or federal legislation. It is yet to be determined whether the current sub-national legislation in Africa is well enforced and whether it will lead to the enactment of strong national legislation.

Other African countries like Uganda do not have comprehensive national tobacco control legislation but instead have legislation specifically banning smoking in public places covering the whole country. Uganda’s smoke-free legislation was a result of public interest litigation made possible by the country’s favourable legal system that allows, under Article 50 of the 1995 Constitution of Uganda, any individual or organization (whether aggrieved or not) to bring an action against the violation of another person’s or group’s human right. The High Court held that smoking in public places violated the rights of non-smoking members of the public and ordered the National Environment Management Authority (NEMA) to put in place regulations banning smoking in public places [29,30]. Uganda’s case illustrates the power of human rights and public interest litigation to achieve tobacco control legislation and provides another creative lesson for the advancement of smoke-free policies in African countries, particularly those whose legal systems have favourable constitutional provisions regarding the right to sue (also known as “standing” or “*locus standi*”). Uganda’s smoke-free regulations, however, are poorly enforced, highlighting the need for sustained public awareness campaigns prior to, and after the introduction of a new smoke-free law in order for it to gain public support and become self enforcing [31].

#### 3.1.2. Obstacles to FCTC Implementation

While litigation is a powerful tool to achieve smoke-free policies, the tobacco industry has used it in their attempt to interfere with or block smoke-free progress. In Uganda, British American Tobacco (BAT)’s former quality controller unsuccessfully appealed against the ban on public smoking [32]. The tobacco industry in Kenya went to court to challenge the Tobacco Control Act of 2007 and led to the suspension of the public smoking ban [33]. The past interference of the tobacco industry in tobacco control policies in Kenya (through the actual drafting of tobacco control laws [34] and through the lobbying of policy makers [35]) makes the weaknesses present in the law unsurprising. Kenya’s smoke-free law does not fully meet the FCTC requirements as it allows for specially designated smoking areas [36].

Another obstacle that African countries face in their efforts towards 100% smoke-free policies and legislation is the tobacco industry’s interference in the form of promoting voluntary regulation so as to avert strict regulation through legislation. One such example is the “Courtesy of Choice” program—an initiative promoted by the tobacco industry to urge the hospitality industry to self-regulate and accommodate smokers and non-smokers by providing a choice of non-smoking and smoking areas in the same establishment with the aim of preventing restrictive smokefree legislation. The initiative has since the early 1990’s been launched and promoted by British American Tobacco across the African continent [37].

### 3.2. Packaging and Labeling of Tobacco Products (FCTC Article 11)

#### 3.2.1. Current Status of Legislation and Compliance with the FCTC

Article 11 of the Framework Convention on Tobacco Control, requires a Party to adopt and implement specified effective packaging and labelling measures within three years after the entry into force of the treaty for that Party. Underpinning Article 11 is the FCTC guiding principle that every person should be informed of the health consequences, addictive nature and mortal threat posed by tobacco consumption and exposure to tobacco smoke [38].

According to the 2011 WHO Report on the Global Tobacco Epidemic, only one country in the African region (Mauritius) is in the highest category defined by the report, that is, a country mandating large warnings (average of front and back of the package is at least 50%) and with all 7 appropriate characteristics, namely: specific health warnings mandated; appearing on individual packages as well as on any outside packaging and labelling used in retail sale; describing specific harmful effects of tobacco use on health; are large, clear, visible and legible (e.g., specific colours and font style and sizes are mandated); rotate; include pictures or pictograms; and are written in (all) the principal language(s) of the country [39].

Three countries are in the second category, that is, a country with medium size warnings (average coverage of front and back of package is between 30 and 49%) with all 7 appropriate characteristics or large warnings but missing one or more appropriate characteristics [40]. Eight countries are in the third category, that is a country with medium size warnings but missing some appropriate characteristics or with large warnings but missing four or more appropriate characteristics [41]. Thirty two African countries have no warnings or have small warnings (average coverage of front and back of package is less than 30%) [42].

Although the Seychelles is not placed in the top category in the 2011 WHO Report on the Global Tobacco Epidemic, upon analysis by this paper’s author, its 2009 legislation regarding tobacco health warnings and labelling meets the FCTC Article 11 and its Guidelines (at least 50% of principal area with text, picture or both). However, regulations are yet to be prescribed relating to content of the warnings (text picture or both, rotation, number of warnings and so on) [43]. Mauritius is the only African country with graphic warnings in force. Mauritius’ warnings are the third largest in the world and they cover 60% of the front and 70% of the back (average is 65%) of the principal display areas of the packaging [44]. A recent study in Mauritius found that the eight graphic health warnings were more effective than the text only warnings they replaced [45]. Research like this provides African countries an opportunity to push for graphic warnings as they are shown to be effective.

Effective health warnings and labelling envisioned by the FCTC and the FCTC Article 11 Guidelines are those that better communicate health risks, provoke a greater emotional response and increase the motivation of tobacco users to quit and to decrease their tobacco consumption. Moreover, effectiveness of health warnings increase with their prominence (size, legibility, location) and graphic warnings are particularly effective in communicating health effects among populations with low literacy levels and among youth [46]. It is therefore important that more African countries adopt and implement graphic warnings because of the presence of populations with low literacy levels, high percentage of youth and multiple languages and dialects.

#### 3.2.2. Obstacles to FCTC Implementation

One of the challenges of getting African countries to adopt and implement packaging and labelling requirements including health warning labels that meet the standards of the FCTC and its Guidelines is the popularity of selling single stick cigarettes as opposed to selling them in a complete unopened packet. A similar challenge is the common practice of selling smokeless tobacco in unconventional packaging especially among rural dwellers who may not afford the more expensive cigarettes and among women who due to cultural reasons might prefer to be seen consuming smokeless tobacco products as opposed to cigarettes. African countries can overcome these challenges by addressing them in their legislation, for example, by banning the sale of single stick cigarettes and requiring minimum package size in order to avoid “kiddie packs”, by requiring graphic warnings at points of sale, by sustained sensitisation campaigns highlighting the harms of consumption of smokeless and smoked tobacco products and by requiring health warnings to appear on smokeless tobacco products as well as on smoked tobacco products.

Another obstacle to the implementation of Article 11 of the FCTC and its Guidelines is the interference of the tobacco industry. In Mauritius the tobacco industry carried out delay tactics to undermine the implementation of the law. The industry took advantage of the loophole in the law that did not specify a supply date of the tobacco products that complied with the new law [47]. The tobacco industry therefore stockpiled many tobacco products manufactured before the entry into force of the law and was able to supply the non-compliant tobacco products several months after the law had entered into force. In a later instance, the tobacco industry in Mauritius flouted Mauritius’ packaging and labelling law by selling, in duty free shops, tobacco products that bore no graphic health warnings, had English only texts, had misleading descriptors like “light”, and had tar and nicotine content displayed [48].

### 3.3. Tobacco Advertising, Promotion and Sponsorship (FCTC Article 13)

#### 3.3.1. Current Status of Legislation and Compliance with the FCTC

According to the 2011 WHO Report on the Global Tobacco Epidemic, five countries in the African region are in the highest category defined by the report, that is, have a ban on all forms of direct and indirect advertising [49]. Nineteen countries have a ban on national television, radio and print media as well as on some but not all other forms of direct and/or indirect advertising [50]. Three countries have a ban on national television, radio and print media only [51]. Nineteen countries have either a complete absence of ban, or ban that does not cover national television, radio and print media [52].

Legislative analysis was carried out by the author of this paper, on the laws of the 5 countries that appear in the highest category of the 2011 WHO Report on the Global Tobacco Epidemic (Eritrea, Kenya, Madagascar, Niger and Chad) in order to ascertain whether the laws fully meet the standards of the FCTC Article 13 and Article 13 Guidelines, of a comprehensive ban on all forms of direct and indirect tobacco advertising, promotion and sponsorship [53].

Despite not being a Party to the FCTC, Eritrea’s ban on advertising, promotion and sponsorship meets the FCTC standards as it prohibits indirect and direct tobacco advertising, promotion and sponsorship [54]. It adopts the FCTC definitions of “tobacco advertising and promotion” and “tobacco sponsorship”.

Madagascar also adopts the FCTC definitions of “tobacco advertising and promotion” and “tobacco sponsorship” and bans all forms of tobacco advertising, promotion and sponsorship thereby meeting the standards of the FCTC Article 13 and its Guidelines [55].

Chad jumped from the second highest category in the 2009 WHO Report on the Global Tobacco Epidemic to the top category in the 2011 WHO Report on the Global Tobacco Epidemic as a result of its new 2010 tobacco control law. Chad’s law adopts the FCTC definitions of “tobacco advertising and promotion” and “tobacco sponsorship” and establishes a comprehensive ban on tobacco advertising, promotion and sponsorship, thereby meeting FCTC standards [56].

Niger bans all forms of tobacco advertising, promotion and sponsorship in accordance with the FCTC and also incorporates the FCTC definitions of tobacco advertising and promotion and tobacco sponsorship [57]. According to Article 13 FCTC Guidelines, “national law should enable any interested person or non-governmental organization to initiate legal action against illegal tobacco advertising, promotion and sponsorship” [58]. Accordingly, Niger’s tobacco control law gives tobacco control organisations the right to initiate court proceedings against any violators of the law and in 2007 a tobacco control non-governmental organization—SOS Tabagisme-Niger sued two tobacco companies—BAT-Niger and Sitab for violating the advertising ban [59]. Niger’s example provides an opportunity for civil society to play an important role in the monitoring and enforcement of tobacco control legislation. It also highlights the potential of using the judicial system to advance tobacco control aims such as enforcement of advertising bans.

Kenya’s ban on tobacco advertising promotion and sponsorship is quite broad and includes among other things, banning of tobacco product displays, brand stretching and cross-border promotion [60]. The law bans “sponsoring a sporting, cultural, artistic, recreational, educational or entertainment programme, event or activity” and “sponsoring trade fairs, exhibitions, show or any other events.” [61]. However, Kenya’s law does not have a definition for “tobacco sponsorship” and the law does not prohibit the sponsorship of individuals or organisations. These shortcomings in Kenya's law show that it falls slightly short of meeting the FCTC standards and suggests that it was wrongly categorised in the top grouping in both the 2011 and 2009 WHO Reports on the Global Tobacco Epidemic.

Seychelles is another country that appears to be wrongly categorised in the 2011 and 2009 WHO Reports on the Global Tobacco Epidemic. Although not placed in the top category of the 2011 WHO Report on the Global Tobacco Epidemic, the Seychelles has a complete ban on all direct and indirect forms of domestic and cross border tobacco advertising, promotion and sponsorship [62]. It meets the FCTC and FCTC guidelines standards and should be counted among Africa’s “best practice” countries for FCTC Article 13.

Botswana and South Africa appeared in the top category in the 2009 WHO Report on the Global Tobacco Epidemic, however, due to changes in groupings categorisations or corrections in data, they were relegated to a lower category in the 2011 WHO Report on the Global Tobacco Epidemic. The Botswana law’s definition of “tobacco advertising” is narrower than the definition in the FCTC and it does not expressly prohibit indirect forms of tobacco marketing like brand stretching, tobacco sponsorship and tobacco industry corporate social responsibility schemes [63,64]. Thus Botswana’s law does not fully meet the FCTC and its Guidelines’ standards of a comprehensive ban on all forms of indirect and direct advertising, promotion and sponsorship. In fact, the tobacco industry in Botswana carries out indirect forms of advertising and promotion such as “printing their tobacco product logos on clothing and even worse, school bags, making our children walking billboards” [65].

South Africa’s ban on tobacco advertising, promotion and sponsorship is almost comprehensive. It does does not extend to cross border advertising with regards to books, magazines, newspapers, film or video transmission made outside South Africa [66]. A manufacturer or importer of a tobacco product may make a charitable financial contribution or sponsorship, provided that such contribution or sponsorship is not for the purpose of advertisement [67]. Where the FCTC and its Guidelines calls for a complete ban, the South Africa law provides restrictions on vending machines, product displays at point of sale and point of sale advertising and promotion [68]. However, the law prohibits free distribution of tobacco products, rewards, and gifts for tobacco purchases [69]. The law also bans brand stretching, and tobacco product placement [70]. Furthermore, in order to close the loopholes that the tobacco industry exploited before the 2008 amendments, the law bans “viral”, “buzz” or “guerilla” marketing where industry would promote tobacco *via* text messages and smoking parties or any “organised activity” [70]. In light of this, the tobacco industry (British American Tobacco South Africa) went to court to seek a declaration that the ban on tobacco advertising and promotion is unconstitutional. However, in May 2011 the High Court Judge dismissed the application and ruled that the ban was “reasonable and justifiable in a democratic society” and upheld it. The Judge also dismissed the tobacco industry’s application for an order declaring that the ban did not apply to one-to-one communications between tobacco manufacturers, importers, wholesalers and retailers and consenting adult tobacco consumers [71].

Mauritius’ ban on tobacco advertising, promotion and sponsorship is almost comprehensive. The law’s definitions of “advertise”, and “promote” mirror the FCTC definitions of “tobacco advertising and promotion”. The law adopts the FCTC definition of “tobacco sponsorship” and in effect banning corporate social responsibility schemes of the tobacco industry. However, the tobacco industry has found ways to circumvent this [72]. The law also bans tobacco product displays at point of sale except at duty free shops at the airports of Mauritius and Rodriguez [73,74]. This exemption contravenes the FCTC Article 13 and its Guidelines which call for a total ban on any display and on the visibility of tobacco products at points of sale.

#### 3.3.2. Obstacles to FCTC Implementation

Tobacco industry interference is a major obstacle that African countries face in their efforts to ban tobacco advertising, promotion and sponsorship. A formerly secret industry document belonging to BAT revealed that a “proposed ad ban in Sierra Leone, taken out of the Cabinet at voting stage” was an example of “marketing freedoms in Africa maintained through concerted government relations and effective community involvement programmes” [75]. Fifteen years later, Sierra Leone still has no comprehensive ban on tobacco advertising, promotion and sponsorship.

The tobacco industry also pushes for self-regulation in the area of tobacco advertising, promotion and sponsorship in order to deflect criticism and prevent strict regulation. British American Tobacco was found violating its own voluntary marketing codes in Malawi, Nigeria and Mauritius [76].

Lastly, from the South African example above [71], it is evident that the tobacco industry will use the court system to attempt to block tobacco control legislation and in the case of legislation banning tobacco advertising, promotion and sponsorship, the industry is likely to challenge it on constitutional grounds.

### 3.4. Africa’s Progress in Implementation of the FCTC Articles 8, 11 and 13 can be Summarised as Follows

The African region has shown modest progress in FCTC implementation, with many countries having legislation or policies on the protection from exposure to tobacco smoke, however, only a handful of countries meet the standards of the FCTC Article 8 and its Guidelines particularly with regards to designated smoking areas. Little progress has been achieved on packaging and labelling of tobacco products, with few countries having legislation meeting the minimum standards of the FCTC Article 11 and its Guidelines. Mauritius is the only African country with graphic or pictorial health warnings in place and has the largest warning labels in Africa. Slightly better progress in banning tobacco advertising, promotion and sponsorship has been shown by African countries, although the majority of legislation falls short of the standards of the FCTC Article 13 and its Guidelines. Despite their efforts, African countries’ FCTC implementation at national level has not matched the strong regional commitment and leadership demonstrated during the FCTC treaty negotiations. By not fully implementing the FCTC, many African countries that are Parties to the FCTC stand in breach of their legal obligations under international law to implement the treaty in good faith.

Opportunities to implement the FCTC include the use of litigation, international or regional events, sub-national legislation, and research. Several African countries have used favourable legal systems and political systems to advance tobacco control. The tobacco industry has been a major obstacle to FCTC implementation by employing tactics such as lobbying policy makers, drafting weak texts and use of litigation in order to weaken, delay or block strong tobacco control legislation.

**Figure 2:**
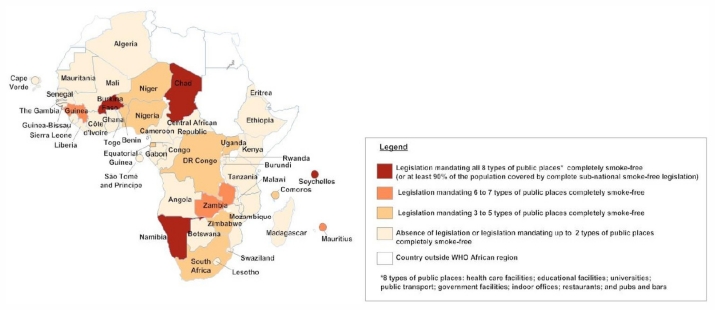
Map of status of FCTC implementation in Africa: Legislation on the protection from exposure to tobacco smoke, as of July 2011.

**Figure 3:**
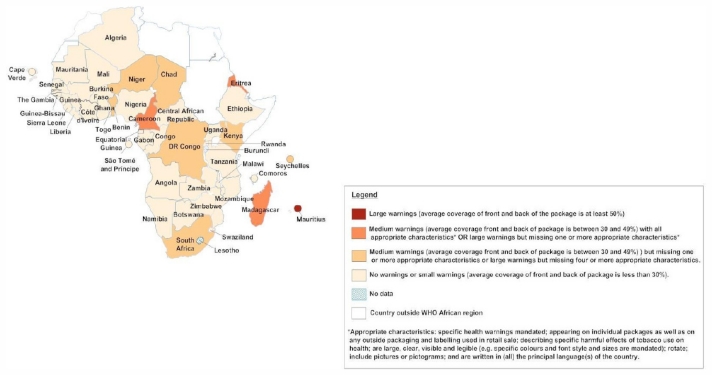
Map of status of FCTC implementation in Africa: Legislation on packaging and labeling of tobacco products, as of July 2011.

**Figure 4:**
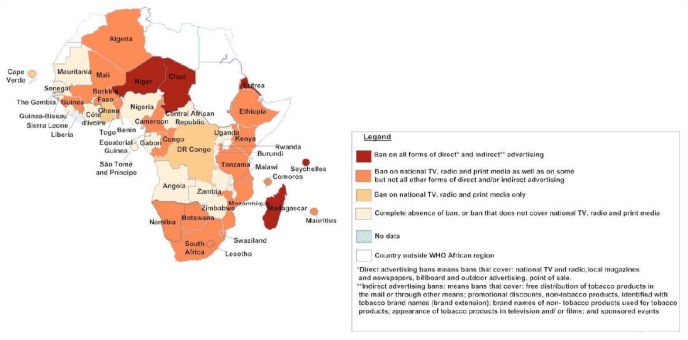
Map of status of FCTC implementation in Africa: Legislation on tobacco advertising, promotion and sponsorship, as of July 2011.

Table 1 and the maps in Figure 2, Figure 3 and Figure 4 show, in graphic form, the status of Africa’s FCTC implementation (Articles 8, 11 and 13).

## 4. Conclusions

This study highlights the need for Africa to step up efforts to adopt and implement effective tobacco control legislation that is fully compliant with the Framework Convention on Tobacco Control. In order to achieve this, countries should prioritise resources for capacity building for drafting strong FCTC compliant legislation, research to inform policy and boost political will, and countering the tobacco industry which is a major obstacle to FCTC implementation in Africa.

## Figures and Tables

**Figure 1:**
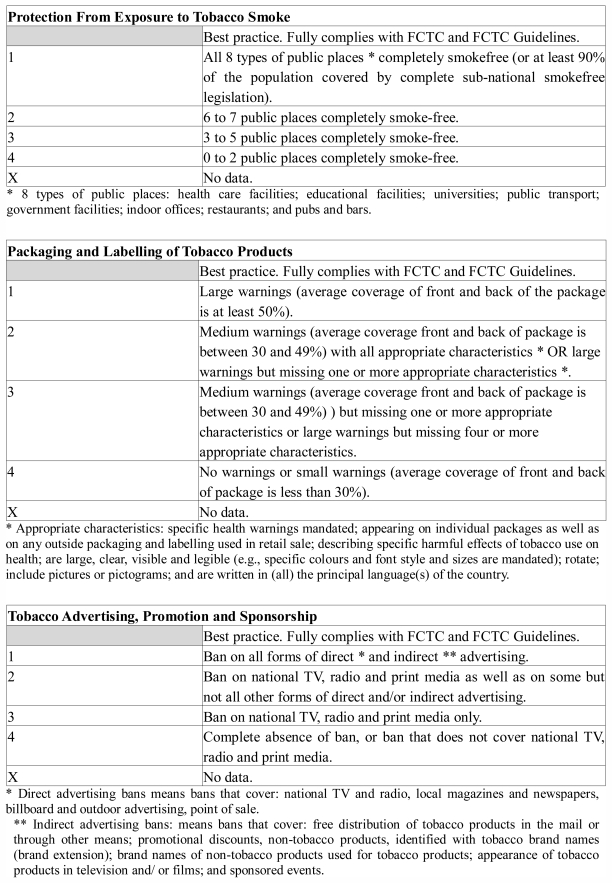
Description of categories in Table 1.

**Table 1:** Status of FCTC implementation in Africa, as of July 2011. *

Country	Protection From Exposure to Tobacco Smoke (Article 8)	Packaging and Labelling of Tobacco Products (Article 11)	Tobacco Advertising, Promotion and Sponsorship (Article 13)
Algeria	4	4	2
Angola	4	4	4
Benin	3	3	2
Botswana	4	4	2
Burkina Faso	1	4	2
Burundi	4	X	4
Cameroon	4	2	2
Cape Verde	4	4	3
Central African Republic	4	4	4
Chad	1	3	1
Comoros	3	4	2
Congo	4	4	2
Cote d ’Ivoire	4	4	4
Democratic Rep of Congo	3	3	3
Equatorial Guinea	3	4	4
Eritrea	4	2	1
Ethiopia	4	4	2
Gabon	4	4	4
Gambia	4	3	2
Ghana	4	4	3
Guinea	2	4	2
Guinea Bissau	4	4	4
Kenya	4	3	2 **
Lesotho	4	X	2
Liberia	4	4	X
Madagascar	4	2	1
Malawi	4	4	4
Mali	4	4	2
Mauritania	4	4	4
Mauritius	2	1	2
Mozambique	4	4	2
Namibia	1	4	2
Niger	3	3	1
Nigeria	3	4	4
Rwanda	4	4	4
Sao Tome and Principe	4	4	4
Senegal	4	4	4
Seychelles	1	3	1 ***
Sierra Leone	4	4	4
South Africa	3	3	2
Swaziland	4	4	2
Togo	4	4	4
United Rep. of Tanzania	4	4	2
Uganda	3 ****	4	4
Zambia	2	4	4
Zimbabwe	3	4	4

Source: reference [77].

* See Figure 1 for description of categories.

** Legislative analysis by the author of this paper, of Kenya’s law relating to tobacco advertising, promotion and sponsorship revealed that it was not in full compliance with FCTC and best practice and thus should have been placed in the second highest category and not the top category in the 2011 Report on the Global Tobacco Epidemic.

*** Legislative analysis by this paper’s authour, of Seychelles’ law relating to tobacco advertising, promotion and sponsorship revealed that it was fully compliant with the FCTC and considered best practice thus should have been placed in the top category and not the second highest in the 2011 WHO Report on the Global Tobacco Epidemic.

**** Uganda was not categorised in the 2011 WHO Report on the Global Tobacco Epidemic. However, if Uganda’s smokefree law had been considered, legislative analysis by the authour of this paper, of the law revealed that it should have been placed in category 3.
